# Improved Information-Theoretic Generalization Bounds for Distributed, Federated, and Iterative Learning †

**DOI:** 10.3390/e24091178

**Published:** 2022-08-24

**Authors:** Leighton Pate Barnes, Alex Dytso, Harold Vincent Poor

**Affiliations:** 1 Department of Electrical and Computer Engineering, Princeton University, Princeton, NJ 08544, USA; 2 Department of Electrical and Computer Engineering, New Jersey Institute of Technology, Newark, NJ 07102, USA

**Keywords:** generalization error, information-theoretic bounds, distribution and federated learning

## Abstract

We consider information-theoretic bounds on the expected generalization error for statistical learning problems in a network setting. In this setting, there are *K* nodes, each with its own independent dataset, and the models from the *K* nodes have to be aggregated into a final centralized model. We consider both simple averaging of the models as well as more complicated multi-round algorithms. We give upper bounds on the expected generalization error for a variety of problems, such as those with Bregman divergence or Lipschitz continuous losses, that demonstrate an improved dependence of 1/K on the number of nodes. These “per node” bounds are in terms of the mutual information between the training dataset and the trained weights at each node and are therefore useful in describing the generalization properties inherent to having communication or privacy constraints at each node.

## 1. Introduction

A key feature of machine learning systems is their ability to generalize new and unknown data. Such a system is trained on a particular set of data but must then perform well even on new data points that have not previously been considered. This ability, deemed generalization, can be formulated in the language of statistical learning theory by considering the generalization error of an algorithm (i.e., the difference between the population risk of a model trained on a particular dataset and the empirical risk for the same model and dataset). We say that a model generalizes well if it has a small generalization error, and because models are often trained by minimizing empirical risk or some regularized version of it, a small generalization error also implies a small population risk, which is the average loss over new samples taken randomly from the population. It is therefore of interest to find an upper bound on the generalization error and understand which quantities control it so that we can quantify the generalization properties of a machine learning system and offer guarantees about its performance.

In recent years, it has been shown that information-theoretic measures such as mutual information can be used for generalization error bounds under the assumption of the tail of the distribution of the loss function [1,2,3,4]. In particular, when the loss function is sub-Gaussian, the expected generalization error can scale at most with the square root of the mutual information between the training dataset and the model weights [2]. Such bounds offer an intuitive explanation for generalization and overfitting: if an algorithm uses only limited information from its training data, then this will bound the expected generalization error and prevent overfitting. Conversely, if an algorithm uses all of the information from its training set, in the sense that the model is a deterministic function of the training set, then this mutual information can be infinite, and there is the possibility of overfitting.

Another modern focus of machine learning systems has been that of distributed and federated learning [5,6,7]. In these systems, data are generated and processed in a distributed network of machines. The main differences between the distributed and centralized settings are the information constraints imposed by the network. There has been considerable interest in understanding the impact of both communication constraints [8,9] and privacy constraints [10,11,12,13] on the performance of machine learning systems, as well as designing protocols that efficiently train the systems under these constraints.

Since both communication and local differential privacy constraints can be thought of as special cases of mutual information constraints, they should pair naturally with some form of information theoretic generalization bounding in order to induce control over the generalization error of the distributed machine learning system. The information constraints inherent to the network can themselves give rise to tighter bounds on generalization error and thus provide better guarantees against overfitting. Along these lines, in a recent work [14], a subset of the present authors introduced the framework of using information theoretic quantities for bounding both the expected generalization error and a measure of privacy leakage in distributed and federated learning systems. The generalization bounds in this work, however, are essentially the same as those obtained by thinking of the entire system, from the data at each node in the network to the final aggregated model, as a single, centralized algorithm. Any improved generalization guarantees from these bounds would remain implicit in the mutual information terms involved.

In this work, we develop improved bounds on the expected generalization error for distributed and federated learning systems. Instead of leaving the differences between these systems and their centralized counterparts implicit in the mutual information terms, we bring analysis of the structure of the systems directly to the bounds. By working with the contribution from each node separately, we are able to derive upper bounds on the expected generalization error that scale with the number of nodes *K* as $O\left(\frac{1}{K}\right)$ instead of $O\left(\frac{1}{\sqrt{K}}\right)$. This improvement is shown to be tight for certain examples, such as learning the mean of a Gaussian distribution with quadratic loss. We develop bounds that apply to distributed systems in which the submodels from *K* different nodes are averaged together, as well as bounds that apply to more complicated multi-round stochastic gradient descent (SGD) algorithms, such as in federated learning. For linear models with Bregman divergence losses, these “per node” bounds are in terms of the mutual information between the training dataset and the trained weights at each node and are therefore useful in describing the generalization properties inherent to having communication or privacy constraints at each node. For arbitrary nonlinear models that have Lipschitz continuous losses, the improved dependence of $O\left(\frac{1}{K}\right)$ can still be recovered but without a description in terms of mutual information. We demonstrate the improvements given by our bounds over the existing information theoretic generalization bounds via simulation of a distributed linear regression example. A preliminary conference version of this paper was presented in [15]. The present paper completes the work by including all of the missing proof details as well as providing new bounds for noisy SGD in Corollary 4.

### Technical Preliminaries

Suppose we have independent and identically distributed (i.i.d.) data $Z_{i}$∼π for i=1,…,n, and let $S=(Z_{1},\ldots ,Z_{n})$. Suppose further that W=A(S) is the output of a potentially stochastic algorithm. Let ℓ(W,Z) be a real-valued loss function and define

$$L\left(w\right)=E_{\pi }\left[\ell \left(w,Z\right)\right]$$

to be the population risk for weights (or model) *w*. We similarly define

$$L_{s}\left(w\right)=\frac{1}{n}\sum_{i=1}^{n}\ell \left(w,z_{i}\right)$$

to be the empirical risk on dataset *s* for model *w*. The generalization error for dataset *s* is then

$$\Delta_{A}\left(s\right)=L\left(A\left(s\right)\right)-L_{s}\left(A\left(s\right)\right)$$

In addition, the expected generalization error is

(1) $$E_{S∼\pi^{n}}\left[\Delta_{A}\left(S\right)\right]=E_{S∼\pi^{n}}\left[L\left(A\left(S\right)\right)-L_{S}\left(A\left(S\right)\right)\right]$$

where the expectation is also over any randomness in the algorithm. Below, we present some standard results for the expected generalization error that will be needed:

**Theorem** **1** (Leave-One-Out Expansion; Lemma 11 in [16]). *Let $S^{\left(i\right)}=\left(Z_{1},\ldots ,Z_{i}^{\prime },\ldots ,Z_{n}\right)$ be a version of S with $Z_{i}$ replaced by an i.i.d. copy $Z_{i}^{\prime }$. Denote $S^{\prime }=\left(Z_{1}^{\prime },\ldots ,Z_{n}^{\prime }\right)$. Then, we have*

$$E_{S∼\pi^{n}}\left[\Delta_{A}\left(S\right)\right]=\frac{1}{n}\sum_{i=1}^{n}E_{S,S^{\prime }}\left[\ell \left(A\left(S\right),Z_{i}^{\prime }\right)-\ell \left(A\left(S^{\left(i\right)}\right),Z_{i}^{\prime }\right)\right]\;.$$

**Proof.**  Observe that

(2) $$E_{S∼\pi^{n}}\left[L\left(A\left(S\right)\right)\right]=E_{S,S^{\prime }}\left[\ell \left(A\left(S\right),Z_{i}^{\prime }\right)\right]$$

for each *i* and that

(3) $$\begin{array}{cc} E_{S∼\pi^{n}}\left[L_{S}\left(A\left(S\right)\right)\right] & =\frac{1}{n}\sum_{i=1}^{n}E_{S∼\pi^{n}}\left[\ell (A\left(S\right),Z_{i})\right]\\ \; & =\frac{1}{n}\sum_{i=1}^{n}E_{S,S^{\prime }∼\pi^{n}}\left[\ell (A\left(S^{\left(i\right)}\right),Z_{i}^{\prime })\right]\;. \end{array}$$

Putting Equations (2) and (3) together with (1) yields the result. □

In many of the results in this paper, we will use one of the two following assumptions:

**Assumption** **1.** *The loss function $\ell (\tilde{W},\tilde{Z})$ satisfies*

$$\log E\left[\exp\left(\lambda \left(\ell \left(\tilde{W},\tilde{Z}\right)-E\left[\ell \left(\tilde{W},\tilde{Z}\right)\right]\right)\right)\right]\leq \psi \left(-\lambda \right)$$

*for λ∈(b,0], $\psi \left(0\right)=\psi^{\prime }\left(0\right)=0$, where $\tilde{W}$ and $\tilde{Z}$ are taken independently from the marginals for W and Z, respectively,*

The next assumption is a special case of the previous one with $\psi \left(\lambda \right)=\frac{R^{2}\lambda^{2}}{2}\;$:

**Assumption** **2.** *The loss function $\ell (\tilde{W},\tilde{Z})$ is sub-Gaussian with parameter $R^{2}$ in the sense that*

$$\log E\left[\exp\left(\lambda \left(\ell \left(\tilde{W},\tilde{Z}\right)-E\left[\ell \left(\tilde{W},\tilde{Z}\right)\right]\right)\right)\right]\leq \frac{R^{2}\lambda^{2}}{2}\;.$$

**Theorem** **2** (Theorem 2 in [3]). *Under Assumption 1, we have*

$$E_{S∼\pi^{n}}\left[\Delta_{A}\left(S\right)\right]\leq \frac{1}{n}\sum_{i=1}^{n}\psi^{*-1}\left(I\left(W;Z_{i}\right)\right)$$

where $\psi^{*-1}\left(y\right)=\inf_{\lambda \in [0,b)}\left(\frac{y+\psi (\lambda )}{\lambda }\right)\;.$

For a continuously differentiable and strictly convex function $F:R^{m}\to R$, we define the associated Bregman divergence [17,18] between two points $p,q\in R^{m}$ to be

$$D_{F}\left(p,q\right)=F\left(p\right)-F\left(q\right)-\langle \nabla F\left(q\right),p-q\rangle \;,$$

where 〈·,·〉 denotes the usual inner product.

## 2. Distributed Learning and Model Aggregation

**Figure 1:**
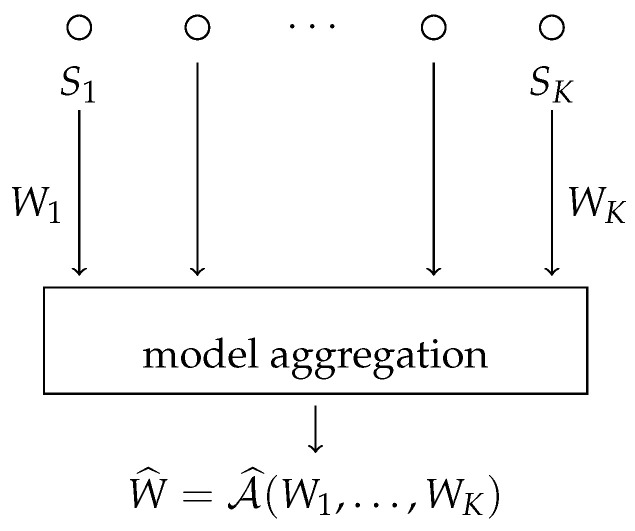
The distributed learning setting with model aggregation.

Now suppose that there are *K* nodes each having *n* samples. Each node k=1,…,K has a dataset $S_{k}=\left(Z_{1,k},\ldots ,Z_{n,k}\right)$, with $Z_{i,k}$ taken i.i.d. from π. We use $S=(S_{1},\ldots ,S_{K})$ to denote the entire dataset of size nK. Each node locally trains a model $W_{k}=A_{k}\left(S_{k}\right)$ with algorithm $A_{k}$. After each node locally trains its model, the models $W_{k}$ are then combined to form the final model $\hat{W}$ using an aggregation algorithm $\hat{W}=\hat{A}\left(W_{1},\ldots ,W_{K}\right)$ (see Figure 1). In this section, we will assume that $W_{k}\in R^{d}$ and that the aggregation is performed by simple averaging (i.e., $\hat{W}=\frac{1}{K}\sum_{k=1}^{K}W_{k}$). Define A to be the total algorithm from the data *S* to the final weights $\hat{W}$ such that $\hat{W}=A\left(S\right)$. In this section, if we say that Assumption 1 or 2 holds, we mean that it holds for each algorithm $A_{k}$. As in Theorem 1, we use $S^{\left(i,k\right)}$ to denote the entire dataset *S* with sample $Z_{i,k}$ replaced by an independent copy $Z_{i,k}^{\prime }$, and similarly, we use $S_{k}^{\left(i\right)}$ to refer to the sub-dataset at node *k*, with sample $Z_{i,k}$ replaced by an independent copy $Z_{i,k}^{\prime }$:

**Theorem** **3.** *Suppose that ℓ(·,z) is a convex function of $w\in R^{d}$ for each z and that $A_{k}$ represents the empirical risk minimization algorithm on local dataset $S_{k}$ in the sense that*

$$W_{k}=A_{k}\left(S_{k}\right)=\underset{w}{\mathrm{argmin}}\sum_{i=1}^{n}\ell \left(w,Z_{i,k}\right)\;.$$

*Then, we have*

$$\Delta_{A}\left(s\right)\leq \frac{1}{K}\sum_{k=1}^{K}\Delta_{A_{k}}\left(s_{k}\right)\;.$$

**Proof.**  (4) $$\begin{array}{cc} \Delta_{A} & \left(s\right)=E_{Z∼\pi }\left[\ell \left(A\left(s\right),Z\right)\right]-\frac{1}{nK}\sum_{i,k}\ell \left(A\left(s\right),z_{i,k}\right)\\ \; & =E_{Z∼\pi }\left[\ell \left(\frac{1}{K}\sum_{k=1}^{K}w_{k},Z\right)\right]-\frac{1}{nK}\sum_{i,k}\ell \left(A\left(s\right),z_{i,k}\right)\\ \; & \leq \frac{1}{K}\sum_{k=1}^{K}E_{Z∼\pi }\left[\ell \left(w_{k},Z\right)\right]-\frac{1}{nK}\sum_{i,k}\ell \left(A\left(s\right),z_{i,k}\right) \end{array}$$

(5) $$\begin{array}{cc} \; & \leq \frac{1}{K}\sum_{k=1}^{K}E_{Z∼\pi }\left[\ell \left(w_{k},Z\right)\right]-\frac{1}{K}\sum_{k=1}^{K}\min_{w}\frac{1}{n}\sum_{i=1}^{n}\ell \left(w,z_{i,k}\right)\\ \; & =\frac{1}{K}\sum_{k=1}^{K}\Delta_{A_{k}}\left(s_{k}\right). \end{array}$$

In the above display, Equation (4) follows by the convexity of *ℓ* via Jensen’s inequality, and Equation (5) follows by minimizing the empirical risk over each node’s local dataset, which exactly corresponds to what each node’s local algorithm $A_{k}$ does. □

While Theorem 3 seems to be a nice characterization of the generalization bounds for the aggregate model (in that the aggregate generalization error cannot be any larger than the average generalization errors over each node), it does not offer any improvement in the expected generalization error that one might expect when given nK total samples instead of just *n* samples. A naive application of the generalization bounds from Theorem 2, followed by the data processing inequality $I\left(\hat{W};Z_{i,k}\right)\leq I\left(W_{k};Z_{i,k}\right)$, runs into the same problem.

### 2.1. Improved Bounds

In this subsection, we demonstrate bounds on the expected generalization error that remedy the above shortcomings. In particular, we would like to demonstrate the following two properties:

(1) The bound should decay with the number of nodes *K* in order to take advantage of the total dataset from all *K* nodes.
(2) The bound should be in terms of the information theoretic quantities $I(W_{k};S_{k})$, which can represent (or be bounded from above by) the capacities of the channels over which the nodes are communicating. This can, for example, represent a communication or local differential privacy constraint for each node.

At a high level, we will improve on the bound from Theorem 3 by taking into account the fact that a small change in $S_{k}$ will only change $\hat{W}$ by a fraction $\frac{1}{K}$ of the amount that it will change $W_{k}$. In the case where *W* is a linear or location model, and the loss *ℓ* is a Bregman divergence, we can obtain an upper bound on the expected generalization error that satisfies properties (1) and (2) as follows:

**Theorem** **4** (Linear or Location Models with Bregman Loss). *Suppose the loss ℓ takes the form of one of the following:*

 *(i)* *$\ell \left(w,\left(x,y\right)\right)=D_{F}\left(w^{T}x,y\right)$;*
 *(ii)* *$\ell \left(w,z\right)=D_{F}\left(w,z\right)$.*

*In addition, assume that Assumption 1 holds. Then, we have*

$$E_{S∼\pi^{nK}}\left[\Delta_{A}\left(S\right)\right]=\frac{1}{K^{2}}\sum_{k=1}^{K}E_{S_{k}∼\pi^{n}}\left[\Delta_{A_{k}}\left(S_{k}\right)\right]$$

*and*

$$\begin{array}{cc} E_{S∼\pi^{nK}}\left[\Delta_{A}\left(S\right)\right] & \leq \frac{1}{nK^{2}}\sum_{i,k}\psi^{*-1}\left(I(W_{k};Z_{i,k})\right)\\ \; & \leq \frac{1}{K^{2}}\sum_{k=1}^{K}\psi^{*-1}\left(\frac{I(W_{k};S_{k})}{n}\right)\;. \end{array}$$

**Proof.**  Here, we restrict our attention to case (ii), but the two cases have nearly identical proofs. Using Theorem 1, we have

$$\begin{array}{cc} \; & E_{S∼\pi^{nK}}\left[\Delta_{A}\left(S\right)\right]\\ \; & =\frac{1}{nK}\sum_{i,k}E_{S,S^{\prime }}\left[\ell \left(A\left(S\right),Z_{i,k}^{\prime }\right)-\ell \left(A\left(S^{\left(i,k\right)}\right),Z_{i,k}^{\prime }\right)\right]\\ \; & =\frac{1}{nK}\sum_{i,k}E_{S,S^{\prime }}[F\left(A\left(S\right)\right)-F\left(Z_{i,k}^{\prime }\right)-\langle \nabla F\left(Z_{i,k}^{\prime }\right),A\left(S\right)-Z_{i,k}^{\prime }\rangle \\ \; & \;\;\;\;\;\;\;-F\left(A\left(S^{\left(i,k\right)}\right)\right)+F\left(Z_{i,k}^{\prime }\right)+\langle \nabla F\left(Z_{i,k}^{\prime }\right),A\left(S^{\left(i,k\right)}\right)-Z_{i,k}^{\prime }\rangle ] \end{array}$$

(6) $$\begin{array}{cc} = & \frac{1}{nK}\sum_{i,k}E_{S,S^{\prime }}\left[\langle \nabla F\left(Z_{i,k}^{\prime }\right),A\left(S^{\left(i,k\right)}\right)-A\left(S\right)\rangle \right]\\ = & \frac{1}{nK}\sum_{i,k}E_{S,S^{\prime }}\left[\langle \nabla F\left(Z_{i,k}^{\prime }\right),\frac{1}{K}W_{k}^{\left(i\right)}+\frac{1}{K}\sum_{j\neq k}W_{j}-\frac{1}{K}\sum_{j}W_{j}\rangle \right] \end{array}$$

(7) $$\begin{array}{cc} = & \frac{1}{nK^{2}}\sum_{i,k}E_{S,S^{\prime }}\left[\langle \nabla F\left(Z_{i,k}^{\prime }\right),W_{k}^{\left(i\right)}-W_{k}\rangle \right]\;. \end{array}$$

In Equation (7), we use $W_{k}^{\left(i\right)}$ to denote $A_{k}\left(S_{k}^{\left(i\right)}\right)$. Equation (6) follows the linearity of the inner product and cancels the higher order terms F(A(S)) and $F(A\left(S^{\left(i,k\right)}\right))$, which have the same expected values. The key step in Equation (7) then follows by noting that $A(S^{\left(i,k\right)})$ only differs from A(S) in the submodel coming from node *k*, which is multiplied by a factor of $\frac{1}{K}$ when averaging all of the submodels. By backing out of Equation (6) and re-adding the appropriate canceled terms, we get

$$E_{S∼\pi^{nK}}\left[\Delta_{A}\left(S\right)\right]=\frac{1}{K^{2}}\sum_{k=1}^{K}E_{S_{k}∼\pi^{n}}\left[\Delta_{A_{k}}\left(S_{k}\right)\right]\;.$$

By applying Theorem 2, this yields

$$E_{S∼\pi^{nK}}\left[\Delta_{A}\left(S\right)\right]\leq \frac{1}{nK^{2}}\sum_{i,k}\psi^{*-1}\left(I(W_{k};Z_{i,k})\right)\;.$$

Then, by noting that $\psi^{*-1}$ is non-decreasing and concave, we have

$$\begin{array}{cc} \frac{1}{nK^{2}}\sum_{i,k} & \psi^{*-1}\left(I(W_{k};Z_{i,k})\right)\leq \frac{1}{K^{2}}\sum_{k=1}^{K}\psi^{*-1}\left(\sum_{i=1}^{n}\frac{I(W_{k};Z_{i,k})}{n}\right)\;. \end{array}$$

Using the property that conditioning decreases entropy yields

$$\sum_{i=1}^{n}I\left(W_{k};Z_{i,k}\right)\leq I\left(W_{k};S_{k}\right)\;,$$

and we have

$$\begin{array}{cc} \frac{1}{K^{2}}\sum_{k=1}^{K} & \psi^{*-1}\left(\sum_{i=1}^{n}\frac{I(W_{k};Z_{i,k})}{n}\right)\leq \frac{1}{K^{2}}\sum_{k=1}^{K}\psi^{*-1}\left(\frac{I(W_{k};S_{k})}{n}\right) \end{array}$$

as desired. □

The result in Theorem 4 is general enough to apply to many problems of interest. For example, if $F\left(p\right)={∥p∥}_{2}^{2}$, then the Bregman divergence $D_{F}$ gives the ubiquitous squared $\ell^{2}$ loss (i.e., $D_{F}\left(p,q\right)={∥p-q∥}_{2}^{2}\;$). For a comprehensive list of realizable loss functions, the interested reader is referred to [19]. Using *F* above, Theorem 4 can be applied to ordinary least squares regression, which we will examine in greater detail in Section 4. Other regression models such as logistic regression have loss functions that cannot be described with a Bregman divergence without the inclusion of additional nonlinearity. However, the result in Theorem 4 is agnostic to the algorithm that each node uses to fit its individual model. In this way, each node could fit a logistic model to its data, and the total aggregate model would then be an average over these logistic models. Theorem 4 would still control the expected generalization error for the aggregate model with the extra $\frac{1}{K}$ factor. However, critically, the upper bound would only be for the generalization error that is with respect to a loss of the form $D_{F}\left(w^{T}x,y\right)$, such as quadratic loss.

In order to show that the dependence on the number of nodes *K* from Theorem 4 is tight for certain problems, consider the following example from [3]. Suppose that *Z*∼$\pi =N(\mu ,\sigma^{2}I_{d})$ and $\ell \left(w,z\right)={∥w-z∥}_{2}^{2}$ so that we are trying to learn the mean μ of a Gaussian distribution. An obvious algorithm for each node to use is simple averaging of its dataset:

$$w_{k}=A_{k}\left(s_{k}\right)=\frac{1}{n}\sum_{i=1}^{n}z_{i,k}\;.$$

For this algorithm, it can be shown that

$$I\left(\hat{W};Z_{i,k}\right)=\frac{d}{2}\log\frac{nK}{nK-1}$$

and

$$\psi^{*-1}\left(y\right)=2\sqrt{d{\left(1+\frac{1}{nK}\right)}^{2}\sigma^{4}y}$$

See Section IV.A. in [3] for further details. If we apply the existing information theoretic bounds from Theorem 2 in an end-to-end way, such as in the approach from [14], we would get

$$\begin{array}{cc} E_{S∼\pi^{nK}}\left[\Delta_{A}\left(S\right)\right] & \leq \sigma^{2}d\sqrt{2{\left(1+\frac{1}{nK}\right)}^{2}\log\frac{nK}{nK-1}}\\ \; & =O\left(\frac{1}{\sqrt{nK}}\right)\;. \end{array}$$

However, for this choice of algorithm at each node, the true expected generalization error can be computed to be

$$E_{S∼\pi^{nK}}\left[\Delta_{A}\left(S\right)\right]=\frac{2\sigma^{2}d}{nK}\;.$$

By applying our new bound from Theorem 4, we get

$$\begin{array}{cc} E_{S∼\pi^{nK}}\left[\Delta_{A}\left(S\right)\right] & \leq \frac{\sigma^{2}d}{K}\sqrt{2{\left(1+\frac{1}{n}\right)}^{2}\log\frac{n}{n-1}}\\ \; & \leq O\left(\frac{1}{K\sqrt{n}}\right) \end{array}$$

which shows the correct dependence on *K* and improves upon the $O\left(\frac{1}{\sqrt{K}}\right)$ result from prior information theoretic methods.

### 2.2. General Models and Losses

In this section, we briefly describe some results that hold for more general classes of models and loss functions, such as deep neural networks and other nonlinear models:

**Theorem** **5** (Lipschitz Continuous Loss). *Suppose that ℓ(w,z) is Lipschitz continuous as a function of w in the sense that*

$$|\ell \left(w,z\right)-\ell \left(w^{\prime },z\right)|\leq C∥w-w^{\prime }{∥}_{2}$$

*for any z and that $E\left[{∥W_{k}-E\left[W_{k}\right]∥}_{2}\right]\leq \sigma_{0}$ for each k. Then, we have*

$$\begin{array}{cc} E_{S∼\pi^{nK}}\left[\Delta_{A}\left(S\right)\right] & \leq \frac{2C\sigma_{0}}{K}\;. \end{array}$$

**Proof.**  Starting with Theorem 1, we have

$$\begin{array}{cc} E_{S∼\pi^{nK}} & \left[\Delta_{A}\left(S\right)\right]\\ \; & =\frac{1}{nK}\;\sum_{i,k}E_{S,S^{\prime }}\;\left[\ell \left(A\left(S\right),Z_{i,k}^{\prime }\right)-\ell \left(A\left(S^{\left(i,k\right)}\right),Z_{i,k}^{\prime }\right)\right] \end{array}$$

(8) $$\begin{array}{cc} \; & \leq \frac{1}{nK}\sum_{i,k}E_{S,S^{\prime }}\left[C{∥A\left(S\right)-A\left(S^{\left(i,k\right)}\right)∥}_{2}\right]\\ \; & =\frac{1}{nK^{2}}\sum_{i,k}E_{S,S^{\prime }}\left[C{∥W_{k}-W_{k}^{\left(i\right)}∥}_{2}\right] \end{array}$$

(9) $$\begin{array}{cc} \; & \leq \frac{C}{nK^{2}}\sum_{i,k}E_{S,S^{\prime }}\left[{∥W_{k}-E\left[W_{k}\right]∥}_{2}\right]+E_{S,S^{\prime }}\left[{∥W_{k}^{\left(i\right)}-E\left[W_{k}\right]∥}_{2}\right] \end{array}$$

(10) $$\begin{array}{cc} \; & \leq \frac{2C\sigma_{0}}{K}\;, \end{array}$$

where Equation (8) follows from Lipschitz continuity, Equation (9) uses the triangle inequality, and Equation (10) is assumed. □

The bound in Theorem 5 is not in terms of the information theoretic quantities $I(W_{k};S_{k})$, but it does show that the $O\left(\frac{1}{K}\right)$ upper bound can be shown for much more general loss functions and arbitrary nonlinear models.

### 2.3. Privacy and Communication Constraints

Both communication and local differential privacy constraints can be thought of as special cases of mutual information constraints. Motivated by this observation, Theorem 4 immediately implies corollaries for these types of systems:

**Corollary** **1** (Privacy Constraints). *Suppose each node’s algorithm $A_{k}$ is an ε-local, differentially private mechanism in the sense that $\frac{p(w_{k}|s_{k})}{p(w_{k}|s_{k}^{\prime })}\leq e^{\varepsilon }$ for each $w_{k},s_{k},s_{k}^{\prime }$. Then, for losses ℓ of the form in Theorem 4, and under Assumption 2, we have*

$$E_{S∼\pi^{nK}}\left[\Delta_{A}\left(S\right)\right]\leq \frac{1}{K}\sqrt{\frac{2R^{2}\min\left\{\varepsilon ,\left(e-1\right)\varepsilon^{2}\right\}}{n}}\;.$$

**Proof.**  Note that

$$\begin{array}{cc} I(W_{k};S_{k}) & =\sum_{w_{k},s_{k}}p\left(w_{k},s_{k}\right)\log\frac{p(w_{k}|s_{k})}{\sum_{s_{k}^{\prime }}p\left(w_{k}|s_{k}^{\prime }\right)p\left(s_{k}^{\prime }\right)}\\ \; & \leq \sum_{w_{k},s_{k}}p\left(w_{k},s_{k}\right)\log\frac{p(w_{k}|s_{k})}{\inf_{s_{k}^{\prime }}p\left(w_{k}|s_{k}^{\prime }\right)}\\ \; & \leq \sum_{w_{k},s_{k}}p\left(w_{k},s_{k}\right)\varepsilon =\varepsilon \;. \end{array}$$

Similarly, it is true that

$$\begin{array}{cc} I(W_{k};S_{k}) & =\mathrm{KL}(P_{W_{k}S_{k}}∥P_{S_{k}}P_{W_{k}})\\ \; & \leq \mathrm{KL}\left(P_{W_{k}S_{k}}∥P_{S_{k}}P_{W_{k}}\right)+\mathrm{KL}\left(P_{S_{k}}P_{W_{k}}∥P_{W_{k}S_{k}}\right)\\ \; & =\sum_{w_{k},s_{k}}p\left(w_{k}\right)p\left(s_{k}\right)\left(\frac{p(w_{k}|s_{k})}{p(w_{k})}-1\right)\log\frac{p(w_{k}|s_{k})}{p(w_{k})}\\ \; & \leq \sum_{w_{k},s_{k}}p\left(w_{k}\right)p\left(s_{k}\right)\left(e^{\varepsilon }-1\right)\varepsilon \leq \left(e-1\right)\varepsilon^{2} \end{array}$$

where the last inequality is only true for ε≤1. Putting these two displays together gives $I\left(W_{k};S_{k}\right)\leq \min\left\{\varepsilon ,\left(e-1\right)\varepsilon^{2}\right\}$, and the result follows from Theorem 4. □

**Corollary** **2** (Communication Constraints). *Suppose each node can only transit B bits of information to the model aggregator, meaning that each $W_{k}$ can only take $2^{B}$ distinct possible values. Then, for losses ℓ of the form in Theorem 4, and under Assumption 2, this yields*

$$E_{S∼\pi^{nK}}\left[\Delta_{A}\left(S\right)\right]\leq \frac{1}{K}\sqrt{\frac{2\left(\log2\right)R^{2}B}{n}}\;.$$

**Proof.**  The corollary follows immediately from Theorem 4 and

$$I\left(W_{k};S_{k}\right)\leq H\left(W_{k}\right)\leq \left(\log2\right)B\;.$$

□

## 3. Iterative Algorithms

We now turn to considering more complicated multi-round and iterative algorithms. In this setting, after *T* rounds, there is a sequence of weights $W^{\left(T\right)}=\left(W^{1},\ldots ,W^{T}\right)$, and the final model ${\hat{W}}_{T}=f_{T}\left(W^{\left(T\right)}\right)$ is a function of that sequence, where $f_{T}$ gives a linear combination of the *T* vectors $W^{1},\ldots ,W^{T}$. The function $f_{T}$ could represent, for example, averaging over the *T* iterates, choosing the last iterate $W^{T}$ or some weighted average over the iterates. For each round *t*, each node *k* produces an updated model $W_{k}^{t}$ based on its local dataset $S_{k}$ and the previous timestep’s global model $W^{t-1}$. The global model is then updated via an average over all *K* updated submodels:

$$W^{t}=\frac{1}{K}\sum_{k=1}^{K}W_{k}^{t}\;.$$

The particular example that we will consider is that of a distributed SGD, where each node constructs its updated model $W_{k}^{t}$ by taking one or more gradient steps starting from $W^{t-1}$ with respect to random minibatches of its local data. Our model is general enough to account for multiple local gradient steps, as are used in so-called federated learning [5,6,7], as well as noisy versions of SGDs, such as in [20,21]. If only one local gradient step is taken for each iteration, then the update rule for this example could be written as

(11) $$W_{k}^{t}=W^{t-1}-\eta_{t}\nabla_{w}\ell \left(W^{t-1},Z_{t,k}\right)+\xi_{t}$$

where $Z_{t,k}$ is a data point (or minibatch) sampled from $S_{k}$ on timestep *t*, $\eta_{t}$ is the learning rate, and $\xi_{t}$ is some potential added noise. We assume that the data points $Z_{t,k}$ are sampled without replacement so that the samples are distinct across different values of *t*. We will also assume, for notational simplicity, that ${\hat{W}}_{T}=W^{T}$, although the more general result follows in a straightforward manner.

For this type of iterative algorithm, we will consider the following timestep-averaged empirical risk quantity:

$$\frac{1}{KT}\sum_{t=1}^{T}\sum_{k=1}^{K}\ell \left(W^{t},Z_{t,k}\right)\;,$$

and the corresponding generalization error, expressed as

(12) $$\Delta_{\mathrm{sgd}}\left(S\right)=\frac{1}{T}\sum_{t=1}^{T}\left(E_{Z∼\pi }\left[\ell \left(W^{t},Z\right)\right]-\frac{1}{K}\sum_{k=1}^{K}\ell \left(W^{t},Z_{t,k}\right)\right)\;.$$

Note that Equation (12) is slightly different from the end-to-end generalization error that we would get from considering the final model $W^{T}$ and whole dataset *S*. It is instead an average over the generalization error we would get from each model, stopping at iteration *t*. We perform this so that when we apply the leave-one-out expansion from Theorem 1, we do not have to account for the dependence of $W_{k}^{t}$ on past samples $Z_{t^{\prime },k^{\prime }}$ for $t^{\prime }<t$ and $k^{\prime }\neq k$. Since we expect the generalization error to decrease as we use more samples, this quantity should result in a more conservative upper bound and be a reasonable surrogate object to study. The next bound follows as a corollary to Theorem 4:

**Corollary** **3.** *For losses ℓ of the form in Theorem 4, and under Assumption 2 (for each $W_{k}^{t}$), we have*

$$E\left[\Delta_{\mathrm{sgd}}\left(S\right)\right]\leq \frac{1}{T}\sum_{t=1}^{T}\frac{1}{K^{2}}\sum_{k=1}^{K}\sqrt{2R^{2}I\left(W_{k}^{t};Z_{t,k}\right)}\;.$$

In the particular example described in Equation (11), where Gaussian noise $\xi_{t}∼N\left(0,I_{d}\sigma_{t}^{2}\right)$ is added to each iterate, Corollary 3 yields the following. As in [20], we assume that the updates are magnitude-bounded (i.e., $\sup_{w,x}{∥\nabla_{w}\ell \left(w,z\right)∥}_{2}\leq L$), the stepsizes satisfy $\eta_{t}=\frac{c}{t}$ for a constant c>0, and that $\sigma_{t}=\sqrt{\eta_{t}}$:

**Corollary** **4.** *Under the assumptions above, we have*

$$E\left[\Delta_{\mathrm{sgd}}\left(S\right)\right]\leq \frac{2RL}{K}\sqrt{\frac{c}{T}}\;.$$

**Proof.**  The mutual information terms in Corollary 3 satisfy

(13) $$\begin{array}{cc} I(W_{k}^{t};Z_{t,k}) & \leq I(W_{k}^{t},W^{t-1};Z_{t,k}) \end{array}$$

(14) $$\begin{array}{cc} \; & =I\left(W_{k}^{t};Z_{t,k}|W^{t-1}\right)+I\left(W^{t-1};Z_{t,k}\right) \end{array}$$

(15) $$\begin{array}{cc} \; & =I(W_{k}^{t};Z_{t,k}|W^{t-1}) \end{array}$$

(16) $$\begin{array}{cc} \; & \leq \frac{d}{2}\log\left(1+\frac{\eta_{t}^{2}L^{2}}{d\sigma_{t}^{2}}\right) \end{array}$$

(17) $$\begin{array}{cc} \; & \leq \frac{\eta_{t}^{2}L^{2}}{2\sigma_{t}^{2}}=\frac{cL^{2}}{2t}\;. \end{array}$$

Equation (13) follows from the data-processing inequality, Equation (14) is the chain rule for mutual information, and Equation (15) follows from the independence of $Z_{t,k}$ and $W^{t-1}$. Equation (16) is due to the capacity of the additive white Gaussian noise channel, and Equation (17) just uses the approximation log(1+x)≤x. Thus, we have

$$E\left[\Delta_{\mathrm{sgd}}\left(S\right)\right]\leq \frac{1}{TK}\sum_{t=1}^{T}RL\sqrt{\frac{c}{t}}\leq \frac{2RL}{K}\sqrt{\frac{c}{T}}\;.$$

□

## 4. Simulations

**Figure 2:**
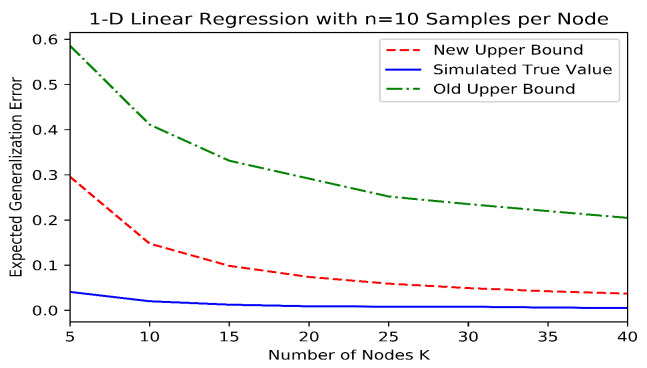

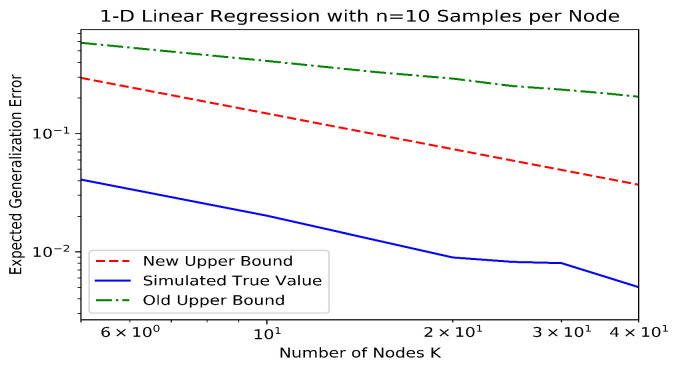
Information-theoretic upper bounds and expected generalization error for a simulated linear regression example in linear (**top**) and log (**bottom**) scales.

We simulated a distributed linear regression example in order to demonstrate the improvement in our bounds over the existing information-theoretic bounds. To accomplish this, we generated n=10 synthetic datapoints at each of *K* different nodes for various values of *K*. Each datapoint consisted of a pair (x,y), where $y=xw_{0}+n$ with x,n∼N(0,1), and $w_{0}$∼N(0,1) was the randomly generated true weight that was common to all datapoints. Each node constructed an estimate ${\hat{w}}_{k}$ of $w_{0}$ using the well-known normal equations which minimize the quadratic loss (i.e., ${\hat{w}}_{k}={\mathrm{argmin}}_{w}\sum_{i=1}^{n}{\left(wx_{i,k}-y_{i,k}\right)}^{2}$). The aggregate model was then the average $\hat{w}=\frac{1}{K}\sum_{k=1}^{K}{\hat{w}}_{k}$. In order to estimate the old and new information-theoretic generalization bounds (i.e., the bounds from Theorems 2 and 4, respectively), this procedure was repeated $M=10^{6}$ times, and the datapoint and model values were binned in order to estimate the mutual information quantities. The value of *M* was increased until the mutual information estimates were no longer particularly sensitive to the number and widths of the bins. In order to estimate the true generalization error, the expectations for both the population risk and the dataset were estimated by Monte Carlo experimentation, with $10^{4}$ trials each. The results can be seen in Figure 2, where it is evident that the new information theoretic bound is much closer to the true expected generalization error and decays with an improved rate as a function of *K*.

## Data Availability

Not applicable.
